# The Antimicrobial Resistance Crisis: Causes, Consequences, and Management

**DOI:** 10.3389/fpubh.2014.00145

**Published:** 2014-09-16

**Authors:** Carolyn Anne Michael, Dale Dominey-Howes, Maurizio Labbate

**Affiliations:** ^1^School of Medical and Molecular Biosciences, University of Technology, Sydney, NSW, Australia; ^2^Asia – Pacific Natural Hazards Research Group, School of Geosciences, University of Sydney, Sydney, NSW, Australia; ^3^ithree Institute, University of Technology, Sydney, NSW, Australia

**Keywords:** antibiotic, antimicrobial resistance, global crisis, horizontal gene transfer, public perception

## Abstract

The antimicrobial resistance (AMR) crisis is the increasing global incidence of infectious diseases affecting the human population, which are untreatable with any known antimicrobial agent. This crisis will have a devastating cost on human society as both debilitating and lethal diseases increase in frequency and scope. Three major factors determine this crisis: (1) the increasing frequency of AMR phenotypes among microbes is an evolutionary response to the widespread use of antimicrobials; (2) the large and globally connected human population allows pathogens in any environment access to all of humanity; and (3) the extensive and often unnecessary use of antimicrobials by humanity provides the strong selective pressure that is driving the evolutionary response in the microbial world. Of these factors, the size of the human population is least amenable to rapid change. In contrast, the remaining two factors may be affected, so offering a means of managing the crisis: the rate at which AMR, as well as virulence factors evolve in microbial world may be slowed by reducing the applied selective pressure. This may be accomplished by radically reducing the global use of current and prospective antimicrobials. Current management measures to legislate the use of antimicrobials and to educate the healthcare world in the issues, while useful, have not comprehensively addressed the problem of achieving an overall reduction in the human use of antimicrobials. We propose that in addition to current measures and increased research into new antimicrobials and diagnostics, a comprehensive education program will be required to change the public paradigm of antimicrobial usage from that of a first line treatment to that of a last resort when all other therapeutic options have failed.

## Introduction

The microbial world, being the sum of all of those organisms that are too small to be discerned with the human eye, is diverse, abundant, and ubiquitous. Containing not only bacteria and viruses but also vast numbers of many different types of multicellular organisms (1, 2), the microbial world is also the basis of the global ecology. These apparently invisible organisms inhabit all ecological niches on this planet, including every surface, cavity, and cellular milieu of every human. The majority of these microbial “passengers” are largely benign or even beneficial to their human hosts through their interactions with the wider ecology. However, some few are active predators, causing damage, morbidity, and even lethal outcomes. The gross effect of these pathogens is infectious disease. While a small proportion of the overall microbial diversity, pathogens are nevertheless numerous and diverse and have evolved many ways of both reaching and then taking advantage of the biological resource represented by their human prey.

In order to combat infectious disease, a suite of chemicals known as antimicrobial agents that are effective in limiting, preventing, or eliminating the growth of microbial predators has been developed. The majority of these antimicrobials originated in natural products where they were originally used by various organisms to defend against microbial attack (3, 4). Having been isolated and characterized, many of these “natural” products have been subsequently modified by humanity to create additional or amplified antimicrobial activity (3). The actions of many of these antimicrobial agents are specific to particular types of pathogen though others may affect broad ranges of microbes. These antimicrobial agents include antiseptics, antibiotics, antifungals, and anti-helminthics, as well as many others targeted against other specific types of pathogen.

The administration of antimicrobials in the treatment and prevention of infectious disease has provoked an evolutionary response among microbes by producing resistance to the applied antimicrobial (5). A graphic example of this is the widespread evolution of antibiotic resistance since the general introduction of penicillin during World War 2. While initially effective against a wide range of bacterial diseases, today, <70 years later, there are increasing numbers of pathogens that are not only resistant to penicillin and its derivatives but also to all other available antibiotics (6). Similarly, many other non-bacterial pathogens such as the causative organism of malaria, *Plasmodium* spp, are now also becoming resistant to all known antimalarial treatments (7). This evolution of resistance in the microbial world is an evolutionary response to the widespread and indiscriminate use of antimicrobials. Combined with the increasingly large and connected human population upon the Earth, prevalent antimicrobial resistance (AMR) will see the rise of not only debilitating but also potentially lethal epidemics for which there is no effective treatment. Such epidemics may be global in extent and ongoing in tenure, and so represent the urgent consequences of the current AMR crisis (8).

In this work, we shall review the causes of the AMR crisis and the current and developmental measures to address it. We also address the likelihood that no solution to this crisis will be definitive, and that continual management of this issue will be required across human society. Finally, we shall propose actions additional to those already being taken to assist in addressing and managing this crisis.

## Consequences of the AMR Crisis to Human Society

In 2013, the Centers for Disease Control (CDC) in the USA asserted that the human race is now in the “post-antibiotic” era (9). In May 2014, the World Health Organization (WHO) stated that the AMR crisis is becoming dire (8). The rise of AMR in current human society will mean that on an individual level, an increasing use of older less effective techniques in controlling infections will be required. Such techniques, including debridement, disinfection, amputation, and isolation will mean that the process of treating infections will take longer, be far more invasive, and will be less successful. While the prospect of global pandemics such as the Spanish Flu of 1918 with their attendant horrendous death tolls are not unlikely, non-lethal illnesses will also extend to more people, more often, and will take longer to resolve. This increasing incidence of debilitating and lethal disease will have a significant effect on human society.

While disease has always been a feature of human society, its probable future impact on a large and increasing human population without the benefit of effective antimicrobials is varied and significant. The economic impact of increasing numbers of untreatable infectious diseases will become significant, as larger numbers of productive individuals are lost from the workforce for increasing periods of time (10, 11). Additionally, the increasing burden of caring for those suffering will place additional loads on their families and community, as well as the wider health care systems. The flow-on effects of this loss of labor and increased load on health services will reduce the national outputs of most countries compared to current levels and will have rippling societal and cultural impacts. In addition to these cumulative effects caused by increasing morbidity, the potential impacts of untreatable, virulent, and lethal pandemics would at least equal the impact of the global Spanish flu epidemic of 1918 where at least 50 million people died (12).

## Why is There an AMR Crisis Now?

Infectious disease is an example of predation by microbial organisms upon macroscopic organisms and has been a feature of the ecology of the planet for eons. Epidemics such the bubonic plague, influenza, and many others have been present throughout history, and yet our current situation is a crisis threatening the ongoing health of humanity that surpasses any previous threat by infectious disease. That this crisis has come to a turning point now is the inevitable co-incidence of a number of factors within the ecology of the planet and humanity’s place within it.

### Microbial causes

The microbial world includes organisms whose direct ancestors were present at the beginnings of life on this planet approximately 3.5 billion years ago. As well as having a long heritage of survival on the Earth, microbes today are both abundant and diverse, with over 10 billion (10e^7^) individuals, typically of thousands of different types, present in a typical gram of soil (1, 2). The success of microbial life and hence larger life on this planet can be linked directly to the microbial ability to rapidly and effectively adapt to environmental change (13, 14). Increasingly, we are becoming aware of the diverse and persistent mechanisms that facilitate this microbial resilience.

Due to their small size, microbes are necessarily simple when compared with large multicellular organisms like humans. This simplicity is reflected in their relatively small complement of genetic material (genome). These small genomes and the individual microbes that carry them cannot each contain the wide variety of accessory genes that might be required to address ever-varying environmental challenges. So, microbes as individuals are limited in their ability to cope with environmental challenges. However, when considered as communities, microbial populations are remarkably protean in their ability to adapt both quickly and effectively. The key to this adaptive strength, lies in the huge numbers of individual microbes present in small volumes, and their rapid generation time, which may be as little as 20 min in the common enteric bacterium *Escherichia coli*. Thus, while random change within a single genome may be rare, the vast microbial numbers present in every environment ensures that variations within populations will occur often and locally. Similarly, the rapid generation time of most microbes ensures that advantageous changes will rapidly become prevalent in the continually growing and evolving microbial community.

One source of genetic variability is mutation. Mutation is the random change in the genetic sequence of an organism and generally has either no effect on the organism or else is deleterious. However, when immense numbers of organisms are involved, the chance of a rare advantageous change arising becomes inevitable. While mutation produces new responses to adaptive challenges, microbial “solutions” to past adaptive challenges have also not been lost. A second source of microbial variability is available in large “reservoirs” of adaptive genes that may be “mobilized” within and between microbial species (13, 15, 16). These mobile adaptive genes are available to entire microbial communities through the varied mechanisms of horizontal gene transfer (HGT). Among those mobile genes that have been so far characterized, many have been shown to provide “solutions” to many different adaptive challenges such as resistance to heavy metals, oxidative stress, UV light, and antibiotic resistance, as well as providing virulence determinants. These adaptive genes may be mobilized both within and between “species” in microbial communities undergoing stress and importantly, it has also been shown that the various mobilizing mechanisms are themselves responsive to stress and mobilize genes more frequently in stressed populations (17). The microbial ability to create new phenotypes and to transfer both novel phenotypes and existing adaptive genes between disparate individuals, rapidly changes the genetic complement and hence, the adaptive ability of not only individuals but also entire microbial communities.

Antimicrobials are designed to either limit or prevent the growth of microbes and in so doing create a selective pressure upon microbial communities. Under antimicrobial treatment only those microbes able to survive and reproduce will predominate within the microbial community, so causing their “advantage” to become common. More aggressive and persistent use of antimicrobials increases the selective pressure on the microbial community to which they are applied, generating more adaptive solutions to the applied stress, faster. The widespread and intense application of antimicrobials by humans provides a strong and polarized selective pressure that will continue to provoke a strong adaptive response in the microbial world.

The depth of microbial history, combined with the demonstrated ability of the microbial world to adapt to even many synthetic compounds suggests that resistance to future antimicrobial strategies is also likely. Consequently, it is likely that the current AMR crisis will not be resolved by a single new product or therapy. However, because the rate at which AMR evolves may be related to the strength of the applied selective pressure, the useful life of existing and prospective therapies may be extended, by significantly moderating the use of both current and forthcoming antimicrobials (18). Accordingly, the rate at which microbial adaptation to antimicrobials occurs *is* amenable to moderation.

### Human causes

#### Human population

According to the United Nations Population Fund (19), there are currently approximately seven billion people upon the Earth and in the surrounding space. The human population passed six billion people in 1999 and is expected to reach eight billion in only a few more years’ time if there is no significant impediment to our growth. Such increases, when graphed across time show the exponential growth that is seen in any organism living in a relatively accommodating environment with abundant resources. Classically seen in bacterial cultures, such growth continues until either resources are exhausted and mass starvation ensues or else the population is limited by other factors.

In addition to the rapidly increasing numbers of humans, we also show a marked propensity for gregarious behavior living in closely associated groups ranging from tens of individuals to many millions. The urbanization of human populations has been a dynamic process that passed a significant milestone in human history early in the twenty-first century. In the year 2007, for the first time more than half the world’s population resided in urban centers (20). Such large numbers of people living in close proximity provides significant opportunity for the rapid proliferation of infectious disease. The need to support such groupings of people requires that agricultural systems are both large and intensive in nature and often in close proximity to population centers, so ensuring additional close links of large groups of humans with the wider biosphere.

In addition to increasing urbanization and increased total population, global humanity is now effectively a single biological population. Individuals may travel to most places on the planet within 1 or 2 days, and may realistically access any other human population and indeed virtually any environment on the planet within a week. This means, firstly, that along with the ability to rapidly transport humans, their attendant microbes and pathogens may also cross the planet rapidly and without significant impediment. Such rapid travel can transport infected individuals across the planet many times before even the first symptoms of infections become apparent, so allowing pathogens to be distributed globally.

The second implication of humanity’s large and effectively contiguous population is that the entire human population is now exposed to both potential and existing pathogens from all environments that humanity comes in contact with. This means that not only are a much broader range of microbial predators given access to human populations but also that those successful predators will have a much larger biological resource to exploit. In short, humanity now presents a large and accessible target for microbial predators across the planet (8).

#### Overuse of antimicrobials

When introduced, antimicrobials were a “silver bullet,” capable of rapidly, and specifically treating infectious disease without undue deleterious side effect upon the patient. This remarkable effectiveness lead to their widespread usage, and a persistent belief among the general public that antimicrobials are universally efficacious and should therefore be applied in the first instance to virtually all ailments. The belief in the universal applicability of antimicrobials has resulted in their exuberant use and so has applied a widespread, strong, and polarized selective pressure on the microbial world. This has resulted in the increasing rates of AMR seen today (21–25). Some of the societal practices that illustrate this profligate use of antimicrobials are now discussed.

### Clinical usage

#### Over prescription

The first source of overuse in clinical practice is the empirical use of antimicrobials by clinicians. This random application of antimicrobials is largely due to the practical shortcomings in rapidly and accurately diagnosing infectious disease, its causative pathogen and perhaps most importantly, the susceptibility of the pathogen to a particular antimicrobial therapy. Such accurate diagnosis currently requires multiple laboratory-based tests that can often take days or even weeks to complete. Understandably, the presentation of a patient with life threatening symptoms requires immediate action. Often, this action takes the form of the simultaneous administration of many different antimicrobials in the hope that one will be useful in controlling the unidentified pathogen. This over-application of antimicrobials usually occurs in extreme cases in hospital-based patients and so is relatively controlled, though the development and implementation of rapid and accurate diagnostics would alleviate this problem (26).

Much more widespread than the empirical treatment of acutely ill patients is the serial application of antimicrobials by general practitioners (27). This situation arises when a patient presents to a practitioner with an ailment possibly due to an infectious pathogen. Rather than always testing for pathogen and antimicrobial sensitivity, the practitioner often prescribes an antimicrobial therapy based upon past experience and local epidemiology. This may be effective in reducing the time to cure from initial presentation given the length of time taken for diagnostic testing to occur if the practitioner has guessed correctly regarding the pathogen. However, often the initially prescribed antimicrobial is not appropriate, so requiring repeated visits and successive courses of different antimicrobials until an effective treatment is found. This process not only fails to effect a prompt cure but also subjects the patient’s microbiota to an intense and repeated selective pressure that encourages and conserves the development of AMR among currently non-pathogenic organisms. This resistance may then be transferred via HGT across many microbial communities and importantly, to previously sensitive pathogenic organisms.

Often, the serial application of antimicrobials is driven by patient demand for an immediate resolution to their illness (28). This can take the form of belligerent patients demanding antimicrobials at one extreme and at the other, over prescription by practitioners to appease patients and so garner repeat business. By lessening the demand for the immediate application of antimicrobials in non-acute patients, the use of appropriate diagnostics will be facilitated and so effective and appropriate prescription of antimicrobials will occur more frequently.

#### Entrenched methodologies

Prescribed antimicrobials are generally applied in a fixed regimen, dictating the dose, rate, and period. Typically, such regimens last 5–7 days, though many have now been extended to 14 days or even longer. The fundamental assumption behind such extended regimes is that high dosages over long periods will eradicate the infecting pathogen from the body. However, recent studies demonstrate that the rates of relapse are not significantly higher in patients where the treatment regime ceases as symptoms diminish, as compared to those taking the full course of treatment (29, 30). By limiting courses of treatment to the minimum dose and period required to achieve a clinical result, the selective pressure on “non-combatant” organisms within the patient and the wider environment will be limited, and hence so will the speed of the overall microbial adaptive response.

### Public perception and behavior

The perception by the lay public of antimicrobials as a quick and effective antidote to the majority of maladies has generated behaviors that effectively circumvent the control of a prescribing physician (31).

#### Hoarding

The practice of not completing prescribed courses of antimicrobial treatment may not materially affect the immediate clinical outcome to the patient. However, where the balance of the course is “hoarded” against a perceived future need, the potential for mis-application of antimicrobial therapy to non-susceptible organisms is significantly increased.

#### Non-prescription purchase

Controlled access to antimicrobials is not global. In many countries, the production and sale of antimicrobials is relatively or completely unregulated. This results in the production of therapeutic materials of extremely variable quality that are available to the public cheaply and in large amounts. Before the advent of the Internet, access of such supplies of antimicrobials to first world countries was restricted to returning travelers. However, online shopping has enabled widespread access to even currently restricted antibiotics such as rifampicin and ciprofloxacin (31).

### Agricultural applications

By treating both stock and crops with antimicrobials, the overall health of the stock and crops is improved and hence the ultimate agricultural yield can be significantly increased. The commercial justification of such measures in producing more and higher quality product quickly is clear, and can be further justified when large sections of the human population continue to experience food insecurity and famine. However, the impact of such a consistent selective pressure applied to the natural environment through the gross application of antimicrobials is also clear. The evidence of increasingly abundant and diverse AMR genes in urban, agricultural, and apparently pristine environments, suggests that these activities combined with the domestic and clinical use of antimicrobials have already had profound effects upon the microbial ecology (22–25).

### Commercial pressures

In addition to the commercial pressure encouraging the agricultural usage of antimicrobials, an even more concerted campaign has been directed at the general public. The premise of this campaign is that humans need to be completely and permanently “clean” at a microscopic level. That is, in order to ensure our continued health and happiness, our skins, mouths, and guts must be routinely cleansed of all microbial life. Additionally, other surfaces that we may come into contact with such as tables and floors must also be routinely disinfected. It is true that in some situations, such as in food preparation and in handling potentially infectious material, precautions against microbial infection are necessary. However, the general and complete eradication of microbes from both domestic surfaces and ourselves is neither necessary nor possible or even beneficial to health. Recent anecdotal evidence suggests that the development of a comprehensively equipped immune system requires exposure to environmental antigens (32). This exposure develops a cohort of specific antibody generating cells that are present throughout life and is also the basis by which vaccination is effective. Normally, such cellular cohorts and the immunities they trigger are generated in younger years and are supplemented with exposure to novel antigens later in life. By limiting the exposure to environmental antigens through the over-insistence on “clean” environments the developing suite of immunities may be limited in both children and adults. As a result, with the increasing incidence of such compromised immune system versatility, less virulent infections are more easily able to cause morbidity and mortality (33).

Effective and diverse marketing campaigns convince the general public of the necessity of overarching cleanliness. The profitable nature of such campaigns has now produced a vast range of antimicrobial products routinely used in society. These products now include antimicrobials in everything from floor cleaners to eye-drops. The diversity of such products in public use contrasts strikingly with the relatively few effective disinfection methods typically used in clinical settings, where reliance on bleach, alcohol, autoclaving, and a relatively few other strong disinfectants for surface cleaning have proven sufficient for even such stringent applications.

### Vaccination reluctance

Vaccination is the process whereby the human immune system is exposed to an antigen associated with a known pathogen, without actual infection by the pathogen. This process produces immune cells within the patient that subsequently recognize the pathogen and so provide a rapid immune response should the patient be exposed later in life. Vaccination programs have been effective in limiting the incidence of infectious disease. Diseases such as smallpox and polio have been virtually eliminated from many human populations, and in the case of smallpox, from the environment as well (34). However, it is important to note that vaccination is protective to the vaccinated individual only. Accordingly, in communities where the incidence of vaccination decreases, un-vaccinated individuals become increasingly subject to the re-emergence of diseases previously held at a low level (35). Such re-emergence leads to an increase in pathogen numbers within the population that may then more readily infect individuals who are not yet vaccinated such as small children, or those who are immunocompromised such as older people and those otherwise susceptible. Currently, increasing numbers of people are declining vaccination for themselves and their children, often for specious reasons (36). In so doing, they not only endanger themselves and their children but also create increasing reservoirs of pathogen within society that may subsequently become antimicrobial resistant.

## Summary

The ability of the microbial world to accommodate all existing and in all likelihood all future antimicrobial therapies, indicates that there will not be a durable solution to the AMR crisis. Similarly, the large and increasing human population dictates that increasing numbers of untreatable infections will have an increasingly deleterious effect on both individuals and society. The ongoing nature of the AMR crisis will therefore require a shift in the general perception of antimicrobials and their use at all levels of society so that the effectiveness of both existing and prospective antimicrobials will be protected and extended for as long as possible. Additionally, current efforts to research and develop new antimicrobial materials, therapies, and diagnostics will need to be supported and extended so as to ensure that novel solutions become available before older therapies become ineffective.

In order to extend the efficacy of current antimicrobials within the human population, their use should be minimized wherever possible. Such restricted usage requires the application only of antimicrobials to which the infecting pathogen is sensitive, applied for the minimum amount of time and in the minimum dose required in order to achieve the desired clinical effect. Further, the elimination products from the use of antimicrobials should as far as possible be prevented from entering the wider environment. To achieve this goal, the continuation of both legislative and professional control will certainly be required. Additionally, the education of both the corporate world and the general public will be required so as to reduce the demand for antimicrobials from society (37). This may be facilitated through consultation and collaboration with relevant stakeholders such as health professionals, the general public, agribusiness, pharmaceutical companies, the media, expert, and legislative bodies.

## How is the Crisis Currently Being Handled?

The existence of the antimicrobial crisis and its importance to the future of the human race is widely acknowledged among peak health care bodies such as the CDC and the WHO (8, 9). Along with acknowledgment of the problem, these bodies provide a number of recommendations that have contributed to actions intended to manage the crisis. These recommendations have resulted in a series of actions and programs designed to address the AMR crisis. These activities fall into two main categories: those that address the prevention of disease so that the need for antimicrobials will be limited and those that provide new or more efficient treatments to either augment or supplant existing antimicrobial therapies. In brief, these measures include disease prevention through the use of vector control, vaccination, public education, clinical education, and legislative action is recommended. Rapid and effective disease management through the use of diagnostics for microbial identification, microbial sensitivity testing to existing antimicrobials to determine appropriate therapies are also advocated (8).

All of these initiatives have been useful in improving the health of their target populations. However, the accelerating incidence of infections resistant to antimicrobial therapy indicates that these measures should be expanded and additional measures implemented if resistant microbial infection is to be contained.

## Discussion

The factors contributing to the origins of the AMR crisis are threefold. These are the increasingly large and connected human population, the protean ability of the microbial world to adapt to environmental challenges and the profligate overuse of antimicrobials by human society. We assume that by modifying any or all of these factors, the crisis may be ameliorated.

The size, extent, and connectedness of the human population are not readily subject to influence. This means, firstly, that humanity will continue to be exposed to virtually all existing and presumptive pathogens. Secondly, the entire microbial ecosystem is potentially subject to the antimicrobials used by human society. Consequently, novel pathogens, arising as zoonoses and through other means are exposed to the global antimicrobial use, and so resistance in these new pathogens will also continue to increase (38).

Importantly, the demonstrated ability of the microbial adaptive mechanisms to provide effective “answers” to all environmental challenges encountered over the last 3 billion years indicates that resistance to current antimicrobials will continue to occur as long as a strong selective pressure is imposed upon microbial populations. Additionally, the ability of microbial adaptation to take advantage of even synthetic materials suggests that resistance to existing future antimicrobial strategies will also arise. Therefore, it is likely that there will be no single long-lasting solution to the problem of resistant infectious disease.

The adaptive ability of microbes provides the necessary flexibility for the microbial world to cope with environmental change, and is in essence the basis of the evolutionary process. The microbial world is an integral part of the global ecology and so its adaptability is ultimately of benefit to the global ecology, including humanity. For this reason, attempts to directly inhibit the broad suite of adaptive mechanisms that enable genetic diversity should be avoided. However, this does not mean that the specific response of microbes to the use of antimicrobials may not be indirectly modulated.

It has been demonstrated that many of the microbial adaptive processes are themselves responsive to the intensity of the selective challenge (17, 18). Consequently, by reducing the exposure of the microbial world to antimicrobials the corresponding selective pressure will be reduced and so will the speed with which AMR arises and is disseminated within the microbial world. Therefore, in view of the ongoing nature of the AMR crisis, we suggest that the management of this crisis will require a twofold approach. Firstly, the search for new antimicrobial materials, therapies combined with rapid and cost effective diagnostics must be continued and expanded into the future. Secondly, measures to limit the exposure of the global microbial community to applied antimicrobials should be taken in order to preserve the effectiveness of current antimicrobial therapies. Both of these strategies can be accomplished.

The third factor causing the AMR crisis and that of human behavior causing the extensive use of antimicrobials is also amenable to change. Currently, educational campaigns regarding AMR have been implemented among clinicians and their governing bodies. These campaigns have been effective in raising the awareness of healthcare professionals to the AMR threat. These campaigns have resulted in some alteration of prescribing practices as well as stewardship programs in many countries to restrict the use of some antimicrobials to that of a last resort treatment in particular patients (39, 40). Legislative and regulatory mechanisms have also been emplaced in many countries to restrict and control the use of some groups of antimicrobials such as antibiotics, with more groups of antimicrobials under discussion (41).

While these measures represent good governance and have been effective in advising professional groups and in limiting the use of specific chemicals in some areas, they have not been comprehensive in alleviating the overall use of antimicrobials. Most antimicrobials remain uncontrolled and their use is often ill advised in light of the current resistance crisis. We propose that additional measures are required to reduce humanity’s overuse of antimicrobials.

The current usage of antimicrobials is driven largely by public perception of their value. The desire for a healthy life has been closely linked in the public mind with the absence of all microbes from both the individual and their proximate environment. This desire has its roots in broad public health campaigns that are themselves responsible for increasing levels of hygiene and consequently decreasing infection rates within society. Unfortunately, these reasonable hygienic practices have been extended into the unnecessary overuse of antimicrobials. This overuse has been further encouraged in the general public by the plethora of antimicrobial containing products now being marketed. The perceived benefits of such products have caused them to proliferate, so adding significantly to the selective pressure on the microbial world. Combined with the widespread prophylactic use of antimicrobials in agriculture, these two areas of antimicrobial usage have provided perhaps the most widespread and intense selective pressure upon the environment (25). Both of these areas of antimicrobial use, if reduced significantly, could provide the useful extension in current antimicrobial effectiveness that is needed until new therapies become available.

Experience shows that attempting to directly legislate or otherwise control public and corporate activities on health care issues has proven difficult. Examples include drawn out battles with the tobacco industry to limit smoking despite overwhelming evidence of the debilitating effect. Similarly, attempts to moderate the current obesity epidemic in the western world, which has its roots in the large amount of energy-dense foods consumed and often supplied by fast food chains, have also met significant resistance. The only successes in these and other campaigns have occurred when public opinion has changed significantly, so making the ruling legislative and market conditions untenable. Accordingly, we suggest that in order to limit broad antimicrobial usage and thereby manage the AMR crisis, the first step should be to investigate stakeholder perceptions about the AMR issue generally in order to then address and inform the public perception of appropriate antimicrobial usage (42).

We suggest that the efficacy of all existing measures to address the AMR crisis will be enhanced if the current perception by the general public of antimicrobials as a “first line” of prophylaxis and treatment for potential pathogens were to become an appreciation that antimicrobials of all sorts were to be used only in selected situations and as a “last line” of defense in infectious disease. If such a paradigm shift was achieved, not only would the selective pressure upon the environment be reduced but also the public pressure upon clinical services to instantly provide antimicrobial treatment for ailments would also diminish. Similarly, the need for ever more assertive legislation would also decrease. The moderation of the perceived need for absolute sterility in the home and elsewhere would alleviate the attraction for antimicrobial containing products, and the insistence on maximal agricultural production through the use of antimicrobials would also be reduced.

Human attitudes in all cultures and at all scales have been intentionally and successfully altered throughout history and often for prosaic reasons. The repeated ability of charismatic leaders to sway entire populations is a case in point. Conversely, many attempts to sway public opinion have been unsuccessful, with numerous marketing and political campaigns failing to meet their desired objectives. In order to systematize this area of endeavor, both the measurement and analysis of the public perception, as well as in the more pragmatic area of changing public perception are the subjects of considerable study (43, 44). Among psychologists, sociologists, and geographers, the assessment of risk and its impact on human society involves both the physical factors that might prompt a dangerous situation, and also the perception of people, both singly and in groups, of the potential of that situation to be “risky.” It has been found that the dichotomy between “real” and “perceived” risk and consequently the propensity or otherwise of a population to adopt behaviors that mitigate risk varies significantly with time, cultural, socio-economic, and other demographic factors (44, 45).

Risk perception and risk behavior as a field of enquiry has evolved rapidly in the last 50 years and has developed a variety of theoretical and methodological approaches that can be applied to specific stakeholders to investigate why they hold the views, attitudes, and perceptions (toward risk) that they do (46). Once the context for and drivers of risk perception and behaviors are understood, it becomes possible to develop targeted and effective education programs aimed at behavioral change (47). These findings have resulted in a variety of empirical methodologies, which, with appropriate examination of ruling attitudes and their causes, can lead to a shift in a society’s perception and consequent actions toward hazardous situations (48–50).

In order to inform and therefore influence public perception on the appropriate use of antimicrobials, a social awareness program that is both diverse and prolonged will be required. Because of the ongoing nature of the AMR crisis, a single short duration initiative is unlikely to achieve the permanent shift in public usage of antimicrobials that is required (39). Additionally, a program both sensitive to local attitudes and responsive to changing cultural environments will need to be implemented. Further, in order to increase the public understanding of the basic message of caution in the use of antimicrobials, multiple complimentary “messages” each reinforcing the basic premise will need to be disseminated. Both primary messages such as “antimicrobials should only be taken when prescribed by your doctor” and complementary messages such as “keeping your immune system ready to combat disease will keep you healthy” will be needed. Such a program will also necessarily use multiple means of information delivery ranging from print and televised media through all of the various electronic media as well as personal contact. Finally, in order to ensure that the program remains effective, ongoing sampling of societal values with regard to antimicrobial usage will also necessarily feature.

The objective of permanently changing public perception of antimicrobial usage is necessary in light of the growing AMR crisis. However, such an initiative will require coordination at local, national, and global levels to ensure a consistent “message” and also culturally appropriate local implementation. Perhaps most importantly, the overall ethical conduct of such a broad based public information campaign must be regularly scrutinized so that the impetus of the program is not lost due to intervening loss of direction or scandal.

The healthcare sector has been largely responsible in most aspects of public education to date, and clinicians certainly have an authoritative and personal relationship with most of the community. However, their generally limited continuous access to the broader community necessitates the inclusion of additional groups to promulgate such a broad based change in public attitude. Such groups might include existing public health organizations such as NGO’s, governmental agencies such as disaster management committees, or even the incorporation of dedicated organizations. However, while such long-term oversights are necessary, the first steps in implementing such measures can be more immediate and accessible. We propose that in the first instance, a broad based study into the current public perception of antimicrobial usage should be implemented immediately. By considering economic, geographic, and ethnographic factors in such a study, useful information in developing a suitable subsequent public education campaign will arise. Moreover, we propose that such a study should not be limited to one locality or even one country. In so doing, the starting point for the following information campaign can be established. Additionally, by bringing together the expertise of various scientists, and their disparate disciplines as well as oversight groups, in commencing such a study, a kernel of specialists in the problem having a common dialog will be created. Such a co-operative multinational approach would yield the basis of a coordinated global approach to the management of the AMR crisis.

## Conclusion

The current AMR crisis is likely to be a permanent feature of human society, causing increased human suffering and attendant social costs. Managing this crisis so as to limit its effect upon humanity will require a fundamental shift in the global perception of antimicrobial usage. We believe that such a shift is certainly possible and that the first steps in achieving this must be taken as soon as possible.

## Conflict of Interest Statement

The authors declare that this work was conducted in the absence of any commercial or financial relationships that could be construed as a potential conflict of interest.
